# Supplementary material for: Mechanically-primed voltage-gated proton channels from angiosperm plants
Source: Nat Commun. 2023 Nov 18;14:7515. doi: 10.1038/s41467-023-43280-5 (PMC10657467; doi:10.1038/s41467-023-43280-5)
Supplement: Supplementary file 1 — Supplementary Information [file 41467_2023_43280_MOESM1_ESM.pdf]

SUPPLEMENTARY INFORMATION

Mechanically-primed voltage-gated proton channels from angiosperm plants

Chang Zhao[1], Parker D. Webster,[1] Alexis De Angeli,[2, @] and Francesco Tombola[1, @]

[1]Department of Physiology and Biophysics, University of California, Irvine, CA 92697, USA
[2]IPSiM, University of Montpellier, CNRS, INRAE, Institut Agro, Montpellier, France
[@]Correspondence: F.T (ftombola@uci.edu), A.D.A. (alexis.deangeli@cnrs.fr)

SUPPLEMENTARY FIGURES S1 – S12

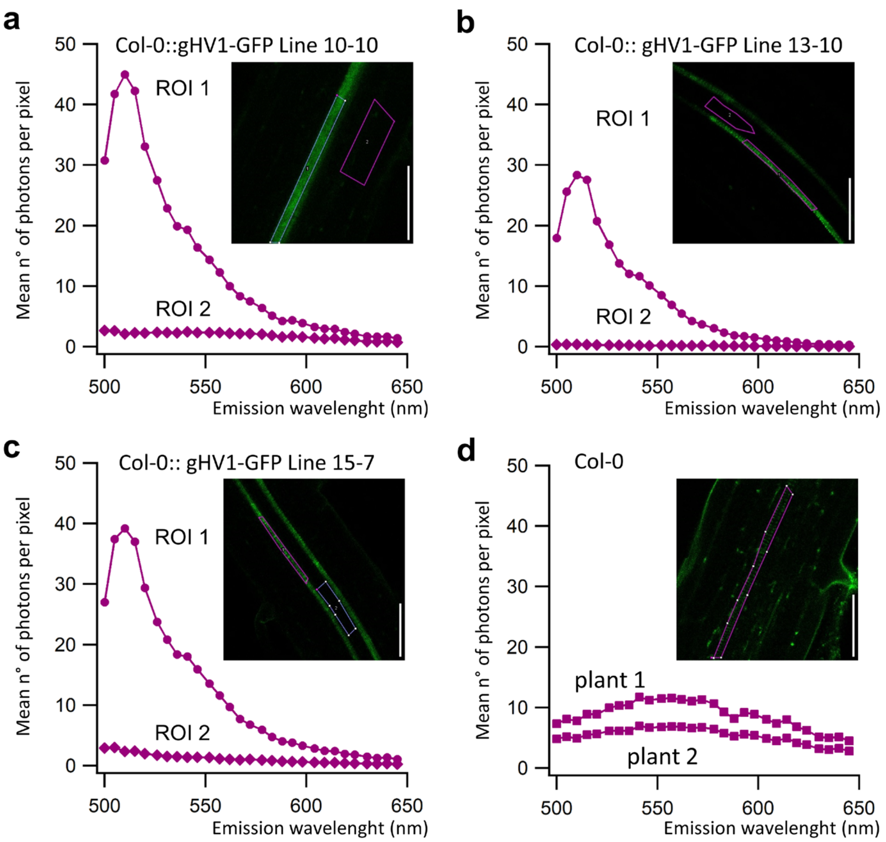

Figure S1. Fluorescence emission spectra recorded in *Arabidopsis thaliana* roots expressing gAtHV1-GFP. a-d) The emission spectral characteristic of the fluorescence (excitation at 488 nm) in the vascular tissues of Arabidopsis roots in the elongation zone was measured in three independent transgenic lines expressing gAtHV1-EGFP (a-c) and two wild-type (Col-0) plants (d). In (a-c), two regions of interests (ROI), one with fluorescent signal (ROI1) and one with background level fluorescence (ROI2), were selected in each root. In all three transgenic lines the emission spectra of ROI1 displayed the behavior of the EGFP with a peak fluorescence at 510 nm. Emission spectra of root vascular tissues of wild-type plants (d) illustrate the background autofluorescence emission properties. The comparison between wild-type and gAtHV1-EGFP expressing plants confirms that the fluorescence detected in the vascular cells of the transgenic lines originates from the EGFP and not from xylem autofluorescence. Scale bar 50 µm.

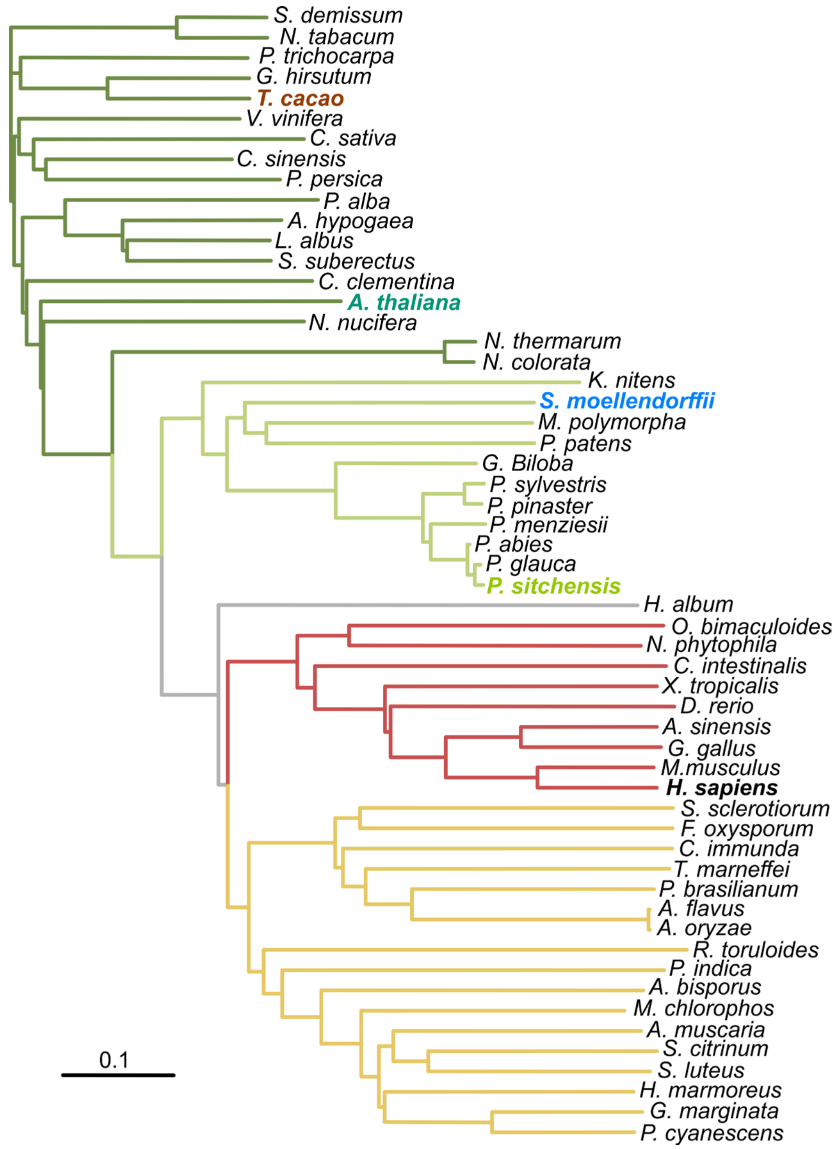

Figure S2. Phylogenetic relationship between Hv channels from plants, fungi, and animals. Organisms from the kingdom *Plantae* includes representatives from flowering (dark green) and non-flowering plants (light green). Animal and fungal branches are shown in red and yellow, respectively. Gray color indicates unranked. Tree scale 0.1 = 10 % difference between sequences. For sequence IDs, see Methods.

```
H.s.   MA--TWDEK-----AVTRRAKVAPAERMSKFLRHFTVVGDDYHAWNINYKKWENEEEEEE   53
P.s.   MGSGTLTDSLLIMDPSSKNQKKNPSSAPPSDL------GGLRDSIELIKEEWQKRRKVVR   54
S.m.   ----------MLLKARKAGQNWR---SKSSTL------GSSRPSGD--------------   27
A.t.   --------------------MN---IINTG----------TVDNVEFSIQNLIKSWCRRR   27
T.c.   ------------MNPTNCTHGPS---PQSVSI------ESVEISIQNLIKSWYKRQRWQH   39

                                             S0                S1
H.s.   EEQPPPTPVSGEEGRAAAPDVAPAPGPAPRAPLDFRGMLRKLFSSHRFQVIIICLVVLDA   113
P.s.   -WWQHLLPVQG------------------CRRAQWRKRLGEFLESTPIHVGTLILLLIDL   95
S.m.   DHESHRDSSSN------------------SSSSSWRHNLRGALESTPAHVTIVALLLIDL   69
A.t.   KWRQLCNFSPKQQ-----------QEELISINQQWRITLSNFLESYQVHLFTIFLLSLDI   76
T.c.   -FFNPRLELVVS-----------------SSRAPWRTHLAKFLESTQLRIVAISLLLLDL   81

         S1                                                   S2
H.s.   LLVLAELILDLKIIQPDK-------------------NNYAAMVFHYMSITILVFFMMEI   154
P.s.   LSTAVDLLKTLHNKSRDLNHCTALLESCQCVSEF--ERSQSIEFLYWVGIVILSLLLFNV   153
S.m.   LATAVDLLLTIHNTSADLRECSASVRACQCDPTGFSSGREPWEFLHWISVGILGVLMLNV   129
A.t.   ILTSLELSSS-------LLSCTSVKK-----------TETENEWFRWGGTVILSILAVKS   118
T.c.   VLTILELSST-------ILSCSPKN----------SSINIDKAWYHWVGISILALLSAKT   124

         S2                S3                           S4
H.s.   IFKLFVFRLEFFHHKFEILDAVVVVVSFILDIVLLFQEHQFEALGLLILLRLWRVARIIN   214
P.s.   GGLLVAFGASFFCHPGYVLDLLVLTTALCLEIFLD-----AQTAGLLVILNLWRIVRVAH   208
S.m.   IGLMVAFGASFFKHAGYVLDLFVVTSALVLEVFFQ-----ADTAGLLIILNLWRIVRVAH   184
A.t.   MALVVAMGKSFFKQPGCVMDGTLAIVALILQVLLE-----KKGTGFIVVVSLWRVLRVVE   173
T.c.   VALAVGLGSAFLRRPGYLVDGIVVVGALLLEALLE-----RKGGGLLAIVSLWRVVRVVE   179

         S4                       CCD
H.s.   GIIISVKTRSERQLLRLKQMNVQLAAKIQHLEFSCSEKEQEIERLNKLLRQHGLLGEVN-   273
P.s.   GIFEVTDDAMESEIHNIEVQFEGLQSKNRDMQELLQQKDQRIAELELEHKTE--------   260
S.m.   GIFEVTDEAWENEIEDMKERIEGAEERFNR---ECGARDRRIQQLEAQLAAAKTQNLDDV   241
A.t.   TAFELSDEAIEVQIDGIISQFQALSKENRTLLETLAEKDEVIKMLEEELNRFKENGDIPF   233
T.c.   SAMELSDEAIEAQIEGIVCQFEALREENTRLLETIAQKDQIIETLEKELDEYRQA-----   234

H.s.   ---    273
P.s.   ---    260
S.m.   L--    242
A.t.   VKP    236
T.c.   ---    234
```

Figure S3. Sequence alignment of four plant Hv channels with the human homolog. Multiple sequence alignment was carried out with Clustal Omega (EMBL-EBI). Highly conserved residues are highlighted. Red asterisk indicates putative selectivity filter aspartate. Green asterisk marks missing first positively-charged residue in the S4 helix of plant Hvs. Blue circles mark residues corresponding to F150 and S181 of hHv1, which are thought to contribute to the binding site for 2GBI and ClGBI in that channel and are not conserved in plant Hvs. Black arrowheads indicate residues of the RSN and outer end of S4 that differentiate flowering from non-flowering plants for the requirement of mechanical priming.

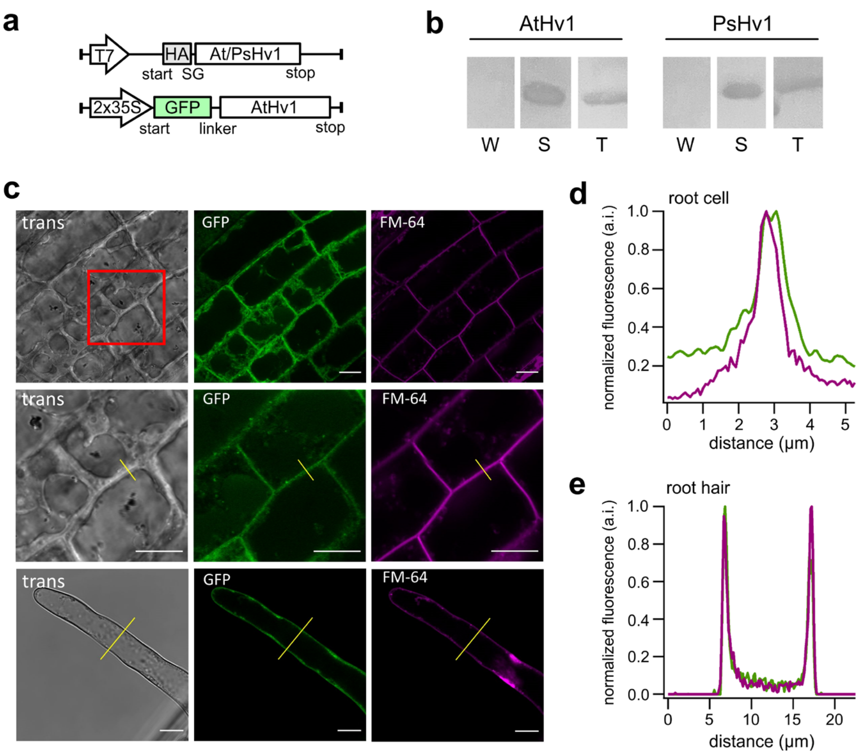

Figure S4. AtHv1 is targeted to the plasma membrane. a) Constructs used to examine protein localization in *Xenopus* oocytes (*top*) and *Arabidopsis* (*bottom*). HA and GFP tags are linked to the N-terminus of the protein via SG and AttB1 linkers, respectively. **b**) Comparison of surface expression of AtHv1 and PsHv1 in *Xenopus* oocytes assessed by pulldown of surface-biotinylated proteins and Western blot. The same number of cells injected with the same amount of mRNA, were processed in parallel for the two channels. W: supernatant before release of bound proteins from streptavidin beads. S: supernatant after release. T: total lysate sample (1:5 dilution). **c**) Subcellular localization of GFP-AtHv1 expressed under the CaMV35s viral promoter in transgenic *Arabidopsis* assessed by confocal fluorescence microscopy (images are representatives of 3 transgenic lines). FM 4-64: plasma membrane marker. Grey-scale images: bright-filed light. Top row: root tip cells. Middle row: magnification of root tip cells (red box). Bottom row: root hair. Scale bar: 10 µm. **d**-**e**) normalized fluorescence quantified along the ROI line (yellow lines in c) of the GFP (green) and FM (magenta) fluorescence in root tip (d) and root hair (e).

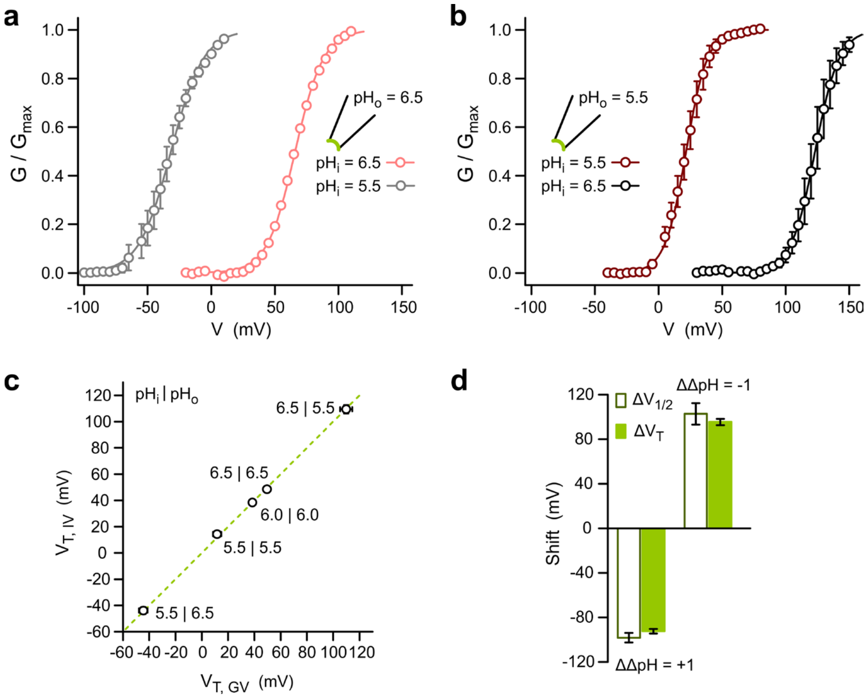

Figure S5. ΔpH dependence of PsHv1 gating quantified using *G*–*V* shifts vs. V_T shifts. a-b) Normalized *G*–*V* relationships for PsHv1 derived from *I*–*V* measurements like those shown in Fig. 3a-b. Each relationship is the mean of n biologically independent experiments, n = 6 (gray), 7 (light red), 5 (dark red), 3 (black). Error bars are SEM. Boltzmann fits are shown in solid lines. $V_{1/2}$ were –32 ± 4 mV, 22 ± 3 mV, 66 ± 1 mV, and 124 ± 4 mV for the following (pH_i|pH_o) conditions: (5.5|6.5), (5.5|5.5), (6.5|6.5), and (6.5|5.5), respectively. Error bars are not shown where smaller than symbols. **c**) Plot correlating V_Ts measured from *I*–*V* curves as in Fig. 3a-d (intercept-method) and V_Ts derived from *G*–*V* curves (see Methods and Source Data). pH conditions are indicated for each point. **d**) ΔpH dependent shifts in voltage range of activation of PsHv1 quantified as $\Delta V_{1/2}$ from *G*–*V* curves compared to corresponding shifts in V_T reported in Fig. 3c. $\Delta V_{1/2}$ mean values were calculate from the following numbers of biologically independent $V_{1/2}$ measurements: n = 6 (5.5|6.5), 7 (6.5|6.5), 5 (5.5|5.5), 3 (6.5|5.5). Error bars are SEM. Source data are provided as a Source Data file.

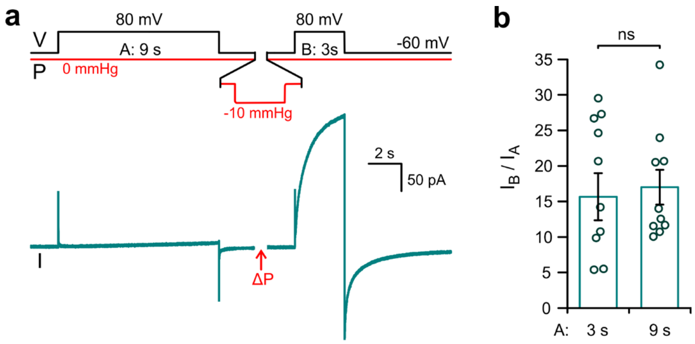

Figure S6. Mechanical priming of AtHv1 assessed with longer initial depolarization. a) Representative proton current elicited by membrane depolarization for AtHv1 before (step A) and after membrane stretch (step B). The stimulation protocol was the same as in Fig. 4b except for the duration of depolarization step A, which was prolonged from 3 s to 9 s. **b**) Averaged increases in current caused by the mechanical stimulus. Current values I_A and I_B were measured at the end of depolarization steps A and B, respectively. Bars represent means of I_B/I_A from n = 10 biologically independent measurements ± SEM. Data for the 3s-long depolarization are the same as shown in Fig. 4c. Welch's t-test was used for statistical analysis. ns: non statistically different. Source data are provided as a Source Data file.

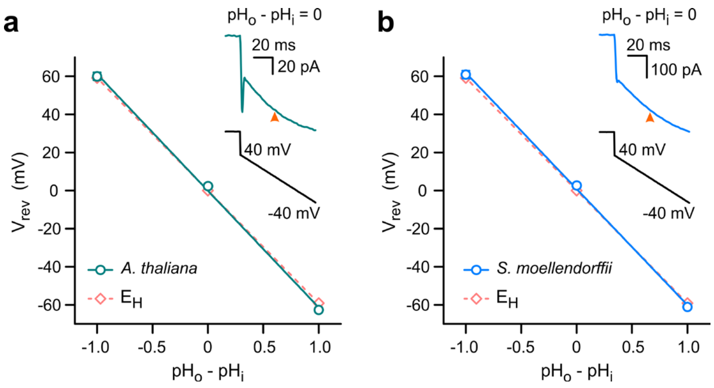

Figure S7. Proton selectivity of AtHv1- and SmHv1-mediated currents after mechanical priming. a-**b**) Reversal potentials of AtHv1 (a) and SmHv1 (b) as a function of ΔpH. Points are means from n biologically independent measurements ± SEM. In (a), n = 5, 13, and 6 for ΔpH = –1.0, 0, and 1.0, respectively. In (b), n = 4, 14, and 5 for ΔpH = –1.0, 0, and 1.0, respectively. Error bars are not shown where smaller than symbols. Slopes of liner fits are –61 ± 2 mV/pH in both cases. The Nernst potential for protons (E_H), is displayed as dashed line (slope: –59 mV/pH unit). ΔpH values of –1, 0, and 1, correspond to the ($pH_i|pH_o$) pairs (6.5|5.5), (5.5|5.5; 6.5|6.5), and (5.5|6.5), respectively. Inserts show examples of V_{rev} measurements from repolarizing voltage ramps following mechanical priming and membrane depolarization (see Methods). In the examples, ΔpH was 0 and the initial depolarization was at 80 mV. Orange arrowheads indicate *I* = 0 pA. Source data are provided as a Source Data file.

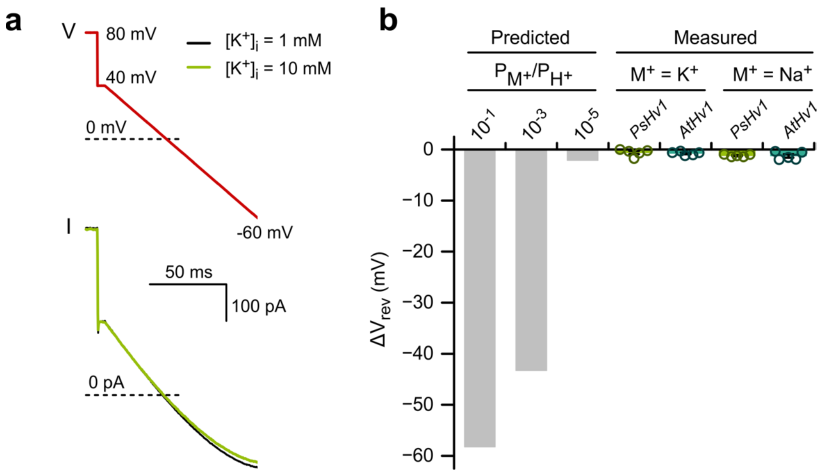

Figure S8. K^+ and Na^+ do not significantly permeate PsHv1 or AtHv1. a) Example of currents (*I*) measured from PsHv1 in response to the indicated voltage ramp (*V*) following a 3s-long depolarization to 80 mV to open the channel. Recordings were performed in inside-out patches under symmetrical pH conditions (pH_i = pH_o = 6.0). Black and green traces were recorded in the presence of 1 mM and 10 mM K^+ in the bath solution, respectively. The K^+ concentration in the pipette was 1 mM. Reversal potentials in the presence and absence of K^+ gradient were used to calculate ΔV_{rev}. **b**) Mean values of ΔV_{rev} for the indicated channels and ions were calculated from n biologically independent recordings like those shown in (a). n = 5 per condition. Error bars are SEM. Mechanical pre-stimulation (ΔP = –10 mmHg for 3 s) was applied to both PsHv1 and AtHv1. Gray bars are ΔV_{rev} values calculated using eq. 5. with the indicated relative permeabilities (P_{M^+}/P_{H^+}). Source data are provided as a Source Data file.

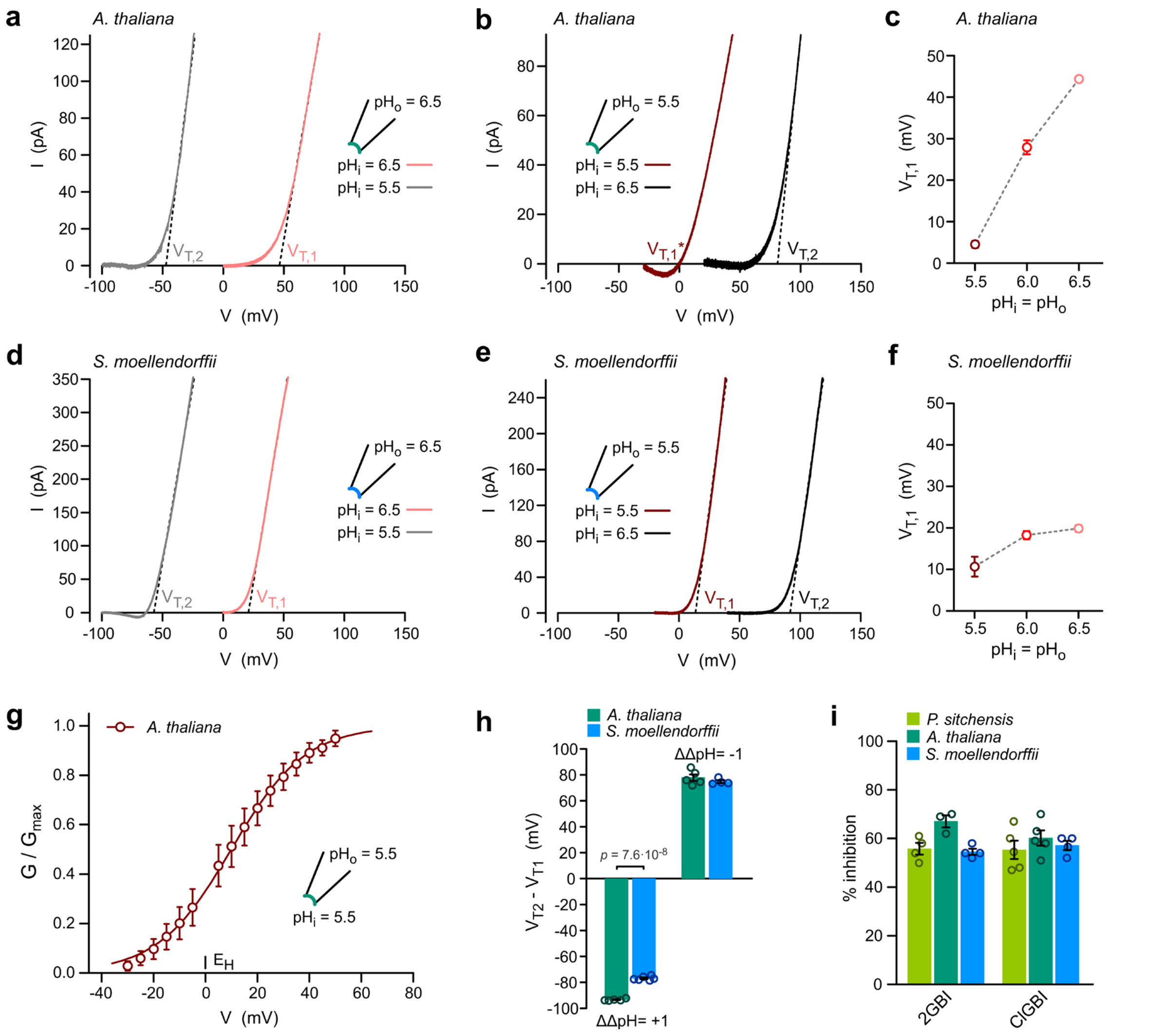

Figure S9. ΔpH dependence and sensitivity to arginine mimics of AtHv1- and SmHv1-mediated currents after mechanical priming. a-b) Changes in *I–V* relationship for AtHv1 in response to changes in ΔpH from 0 to 1 (a) and from 0 to –1 (b). Currents were measured after mechanical priming with voltage ramp protocols described in Methods. **c**) V_{T1} for AtHv1 plotted as a function of pH under symmetrical conditions (ΔpH = 0). Each point represents the mean of n = 5 biologically independent measurements. Error bars (SEM) are not shown where smaller than symbols. **d-e**) same as (a-b) but for SmHv1. **f**) Same as (c) but for SmHv1. Each point represents the mean of n independent measurements ± SEM, n = 4, 3, and 6 for pH = 5.5, 6.0, and 6.5, respectively. **g**) Normalized *G–V* relationship for AtHv1 derived from *I–V* measurements like those shown in (b), dark-red trace. *G–V* represents mean of 5 biologically independent experiments ± SEM. Boltzmann fit is shown in solid lines. $V_{1/2}$ = 10 ± 1 mV, slope = 14.2 ± 0.7. **h**) Average shifts in V_T as a function of change in ΔpH (ΔΔpH) measured from *I–V*s like those shown in (a-b and d-e). Each bar represents the mean of n biologically independent measurements ± SEM. n = 5 (*A.t.*) and 6 (*S.m.*) for pH =1. n = 5 (*A.t.*) and 4 (*S.m.*) for pH = –1. Welch's t-test was used for statistical analysis. **i**) Inhibitions of AtHv1 and SmHv1 by 500 µM 2GBI and 50 µM ClGBI. Currents were measured after mechanical priming in response to depolarization steps to 80 mV, $pH_i = pH_o$ = 6.0 (see Methods). Inhibitions of PsHv1 at the equivalent concentrations of 2GBI and ClGBI (data from Fig. 3e-f) are shown for reference. Each bar represents the average inhibition from n biologically independent measurements ± SEM. n = 4 (*P.s.*), 3 (*A.t.*), and 4 (*S.m.*) for 2GBI. n = 5 (*P.s.*), 5 (*A.t.*), and 4 (*S.m.*) for ClGBI. *At $pH_i = pH_o$ = 5.5, AtHv1 produced substantial inward currents which precluded the measurement of V_{T1} using the V-intercept method. In this case, V_{T1} was calculated from the $V_{1/2}$ and slope parameters of the corresponding *G–V* (panel (g), see Methods). Source data are provided as a Source Data file.

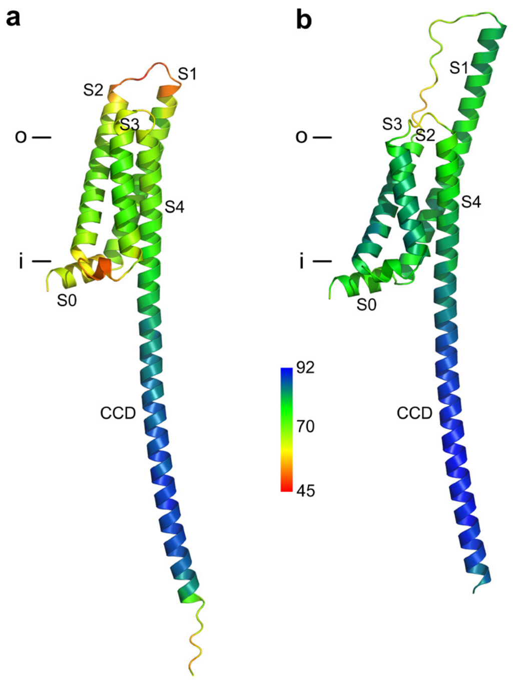

Figure S10. AlphaFold structural models for two plant Hv proteins. a) AtHv1 residues 70 – 260. **b**) PsHv1 residues 51 – 236. Color gradient indicates predicted confidence scores as pLDDT, which varied from a minimum of 45 to a maximum of 92 on a scale of 0 – 100. Regions with pLDDT > 70 are expected to be modeled well.

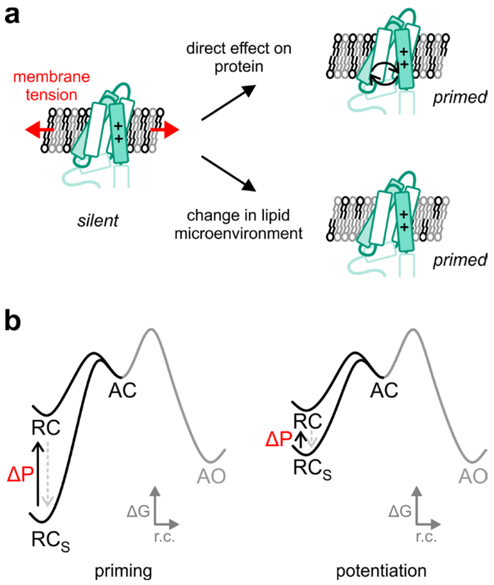

Figure S11. Mechanically-primed voltage-dependent gating a) Two possible mechanisms by which membrane tension could result in AtHv1 priming: 1) membrane tension directly forces the Hv VSD to change conformation, 2) membrane tension causes a change in the lipid microenvironment surrounding the protein, which then causes the Hv VSD to change conformation. **b**) Proposed relationship between mechanical priming of AtHv1 and mechanosensitive potentiation of human Hv1. In AtHv1, channel opening can occur only from the RC state because RC_S is too stable under depolarized conditions (left). The RC_S state of hHv1 could be less stable under the same conditions (right), allowing the channel to open without priming. However, the mechanically-induced transition to the RC state could provide an alternative facilitated pathway to channel opening, resulting in potentiation.

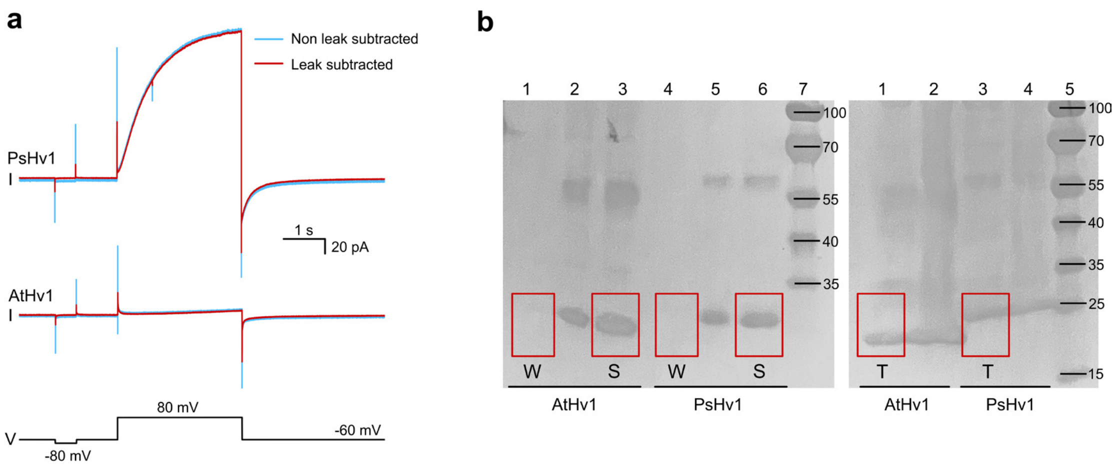

Figure S12. Current traces processing and surface expression evaluation by Western blot. a) Examples of leak subtraction on current traces from the indicated channels before mechanical priming. In some cases (like the ones shown here) a hyperpolarizing pulse preceding the first depolarization was used to aid in the estimation of the leak. Traces without leak subtraction and filtered at 1 kHz are shown in blue. The same traces after leak subtraction, baseline correction, and filtering at 200 Hz are shown in red. **b**) Western blots from which proteins bands shown in Fig. S4b were selected; right: surface proteins, left: cell lysates. Molecular weight marker bands are from Thermo Sci. protein ladder (Cat. # 26617). The additional bands slightly above 55 KDa observed for both plant Hvs are likely due to a multimeric form of the proteins.
