# Supplementary material for: Mechanically-primed voltage-gated proton channels from angiosperm plants
Source: Nat Commun. 2023 Nov 18;14:7515. doi: 10.1038/s41467-023-43280-5 (PMC10657467; doi:10.1038/s41467-023-43280-5)
Supplement: Supplementary file 5 — Reporting Summary [file 41467_2023_43280_MOESM5_ESM.pdf]

# nature portfolio

Corresponding author(s): Francesco Tombola, Alexis De Angeli

Last updated by author(s): Oct 25, 2023

# Reporting Summary

Nature Portfolio wishes to improve the reproducibility of the work that we publish. This form provides structure for consistency and transparency in reporting. For further information on Nature Portfolio policies, see our Editorial Policies and the Editorial Policy Checklist.

## Statistics

For all statistical analyses, confirm that the following items are present in the figure legend, table legend, main text, or Methods section.

| n/a | Confirmed | |
|---|---|---|
| ☐ | ☒ | The exact sample size ($n$) for each experimental group/condition, given as a discrete number and unit of measurement |
| ☐ | ☒ | A statement on whether measurements were taken from distinct samples or whether the same sample was measured repeatedly |
| ☐ | ☒ | The statistical test(s) used AND whether they are one- or two-sided<br>*Only common tests should be described solely by name; describe more complex techniques in the Methods section.* |
| ☒ | ☐ | A description of all covariates tested |
| ☐ | ☒ | A description of any assumptions or corrections, such as tests of normality and adjustment for multiple comparisons |
| ☐ | ☒ | A full description of the statistical parameters including central tendency (e.g. means) or other basic estimates (e.g. regression coefficient) AND variation (e.g. standard deviation) or associated estimates of uncertainty (e.g. confidence intervals) |
| ☐ | ☒ | For null hypothesis testing, the test statistic (e.g. $F$, $t$, $r$) with confidence intervals, effect sizes, degrees of freedom and $P$ value noted<br>*Give P values as exact values whenever suitable.* |
| ☒ | ☐ | For Bayesian analysis, information on the choice of priors and Markov chain Monte Carlo settings |
| ☒ | ☐ | For hierarchical and complex designs, identification of the appropriate level for tests and full reporting of outcomes |
| ☒ | ☐ | Estimates of effect sizes (e.g. Cohen's $d$, Pearson's $r$), indicating how they were calculated |

*Our web collection on statistics for biologists contains articles on many of the points above.*

## Software and code

Policy information about availability of computer code

| | |
|---|---|
| Data collection | pClam10-Clampex, no custom software was used for data acquisition. |
| Data analysis | iTOL 5.6.2, Clustal Omega, MPEx, Coils, AlphaFold v2.1.0, AlphaFold-multimer, PyMOL, Clampfit10.2, Origin v8.1 & v2023, GraphPad Prism 9 |

For manuscripts utilizing custom algorithms or software that are central to the research but not yet described in published literature, software must be made available to editors and reviewers. We strongly encourage code deposition in a community repository (e.g. GitHub). See the Nature Portfolio guidelines for submitting code & software for further information.

## Data

Policy information about availability of data

All manuscripts must include a data availability statement. This statement should provide the following information, where applicable:

- Accession codes, unique identifiers, or web links for publicly available datasets
- A description of any restrictions on data availability
- For clinical datasets or third party data, please ensure that the statement adheres to our policy

All data supporting the findings of this study are available within the paper and its Supplementary Information. The source data underlying Figures 1d, 2b, d, 3c-f, 4c, 5d, and 7d, as well as Fig. S5a-d, S6b, S7a-b, S8b, and S9c-i are provided as a Source Data file. Full image for Fig. S4b is shown in Fig. S12b. Coordinates for structural models shown in Fig. 6 and Fig. S10 are provided in two supplementary PDB files. The Source Data file is provided with this paper.

## Research involving human participants, their data, or biological material

Policy information about studies with human participants or human data. See also policy information about sex, gender (identity/presentation), and sexual orientation and race, ethnicity and racism.

| | |
|---|---|
| Reporting on sex and gender | This study did not involve human participants or human data. |
| Reporting on race, ethnicity, or other socially relevant groupings | Not applicable. |
| Population characteristics | Not applicable. |
| Recruitment | Not applicable. |
| Ethics oversight | Not applicable. |

Note that full information on the approval of the study protocol must also be provided in the manuscript.

# Field-specific reporting

Please select the one below that is the best fit for your research. If you are not sure, read the appropriate sections before making your selection.

[x] Life sciences  [ ] Behavioural & social sciences  [ ] Ecological, evolutionary & environmental sciences

For a reference copy of the document with all sections, see nature.com/documents/nr-reporting-summary-flat.pdf

# Life sciences study design

All studies must disclose on these points even when the disclosure is negative.

| | |
|---|---|
| Sample size | Sample sizes that have provided sufficient statistic significance in same type of studies (ion channel expressed in Xenopus oocytes for electrophysiological measurements) were used. |
| Data exclusions | No data from measurements that passed pre-established quality control were excluded. Standard quality control criteria for electrophysiological measurements were applied. The criteria include current and leak levels, voltage clamp quality, and absence of apparent proton accumulation artifacts. |
| Replication | For electrophysiological measurements, reproducibility was assessed by comparing biologically independent experiments performed over several days or months, and in several cases, by different operators. All attempts at replication were successful. Replicate information is included in the main text and figure legends. For the experiments in plants, results were derived from 3 distinct transgenic lines and, when relevant, 2 distinct control lines. |
| Randomization | The experiments were not randomized. Randomization is not applicable to our study as constructs or plants were members of a group based on their sequence or genotype, respectively. |
| Blinding | The investigators were not blinded to allocation during experiments and outcome assessment. This was inconsequential because the experiments included internal controls and all data were automatically recorded in a way that prevented possible investigator bias to affect the outcome. |

# Reporting for specific materials, systems and methods

We require information from authors about some types of materials, experimental systems and methods used in many studies. Here, indicate whether each material, system or method listed is relevant to your study. If you are not sure if a list item applies to your research, read the appropriate section before selecting a response.

## Materials & experimental systems

| n/a | Involved in the study | |
|---|---|---|
| [ ] | [x] | Antibodies |
| [x] | [ ] | Eukaryotic cell lines |
| [x] | [ ] | Palaeontology and archaeology |
| [x] | [ ] | Animals and other organisms |
| [x] | [ ] | Clinical data |
| [x] | [ ] | Dual use research of concern |
| [ ] | [x] | Plants |

## Methods

| n/a | Involved in the study | |
|---|---|---|
| [x] | [ ] | ChIP-seq |
| [x] | [ ] | Flow cytometry |
| [x] | [ ] | MRI-based neuroimaging |

## Antibodies

| | |
|---|---|
| Antibodies used | HA-tag mouse monoclonal antibody (2-2.2.14) HRP conjugated, Invitrogen/Thermo-Fisher, Lot:XL362093 |
| Validation | Validated by Western Blotting (Thermo-Fisher). |
